# Organo-fluorine chemistry II

**DOI:** 10.3762/bjoc.6.36

**Published:** 2010-04-20

**Authors:** David O'Hagan

**Affiliations:** 1 School of Chemistry, University of St Andrews, Fife, KY16 9ST, United Kingdom

It is a pleasure to be able to introduce this second Thematic Series on ‘organo-fluorine chemistry’ within the *Beilstein Journal of Organic Chemistry*. The series now embeds the subject firmly as a special interest area of the journal. The first series in 2008 presented contributions representing a wide range of organic fluorine chemistry [1–10] and this series continues that trend exploring the synthesis and properties of a new range of organo-fluorine compounds. Contributions have been received from research groups in Australia, China, Germany, Japan, North America, Ukraine and the United Kingdom, representing a particularly international research community.

The introduction of fluorine remains an important specialism in organic chemistry for modulating the physical properties of molecules involved in programmes ranging from bioorganic chemistry to performance materials. Consequently fluorinated organics are of major commercial significance to the pharmaceuticals, agrochemicals, materials and polymer industries with the fluorine fine chemicals industry servicing these industrial sectors. New innovations and insights into the synthesis and the nature and behaviour of organo-fluorine compounds continue to intrigue and this Thematic Series offers a glimpse into the level of activity in the area.

I am delighted that all of the authors have agreed to submit such high quality contributions to render this Thematic Series substantial and make it such a success.

David O'Hagan

St. Andrews, April 2010
